# Characterizing tissue stiffness at the tip of a rigid needle using an opto-mechanical force sensor

**DOI:** 10.1007/s10544-016-0039-1

**Published:** 2016-02-02

**Authors:** S. V. Beekmans, D. Iannuzzi

**Affiliations:** Deparment of Physics and Astronomy and LaserLab Amsterdam, Vrije Universiteit Amsterdam, Amsterdam, The Netherlands

**Keywords:** Ferrule-top technology, In situ indentation, Remote actuation, Interferometry, Tissue Stiffness, Micromechanics, Minimally invasive instrument

## Abstract

We present a novel device that allows the user to measure the Young Modulus of a material at the opening of a 5 mm diameter needle. The device relies on a miniaturized cantilever spring mounted at the end of the needle and interrogated via Fabry-Pérot optical fiber interferometry. The probe is repetitively brought in and out of contact with the sample at the end of the needle by means of a steel cable that is controlled via a piezoelectric actuator located at the proximal end. We demonstrate the ability of our device to detect and quantify layers of varying stiffness during needle insertion in a gelatin phantom and to successfully locate tissue boundaries in bovine liver tissue embedded in gelatin.

## Introduction

The mechanical properties of biological networks are often overseen in functional research of healthy tissue as well as in the diagnosis of potentially diseased tissue and in treatment monitoring. For example, in the classification of skin conditions (such as scars or burn wounds) and the following control of disease progression or healing, physicians in most cases prefer visual assessment and subjective scaling above quantitative mechanical information (Draaijers et al. 2004; Durani et al. 2009; Huang et al. 2004; Gurtner et al. 2008). Moreover, tissue mechanics can be linked to a wide array of physiological processes (Cowin and Doty 2007; Cowin and Humphrey 2007; Butler et al. 2000). Cells have very sophisticated methods to sense and adapt to their mechanical environment. Stem cells, for instance, have been proven to adapt their differentiation to the stiffness of their extracellular matrix (Swift et al. 2013; Fu et al. 2010) and white blood cells, as well as tumor cells, are able to manipulate their own stiffness and shape to migrate in and out of blood vessels (Friedl and Wolf K. 2003). At the tissue level, the interplay between the cellular mechanics and the extracellular network determines the stiffness at the micron scale, changes of which have been associated with Alzheimer’s disease (Murphy et al. 2011) and multiple sclerosis (Wuerfel et al. 2010; Streitberger et al. 2012) (in the brain), cancerous growth (Plodinec et al. 2012; Li et al. 2008) (breast) and osteoarthritis (Stolz et al. 2009; Desrochers et al. 2010) (cartilage). Hence, a method to quantify local mechanical properties of tissue, preferably *in situ*, is of high interest.

Classically, the biomechanical response of complex networks is assessed by means of Atomic Force Microscope (AFM) nanoindentation (Hengsberger et al. 2001; Franze 2011; Zhu et al. 2011; Mathur et al. 2001; Li et al. 2015; Gautier et al. 2015). The utilization of an AFM for classification of biological tissues has, however, some principle limitations that cannot be easily overcome, such as size, stability and flexibility. To mitigate those limitations, we have recently introduced a new probe, called *ferrule-top cantilever*, that provides a good alternative for indentation of biological samples in harsh environments (Chavan et al. 2012; Kahn et al. 2015; Neufurth et al. 2015).

Both AFM and ferrule-top indentation are restricted to probing the surface of a sample, while the advantages of probing in depth (i.e., underneath the surface) would clearly be multiple. By integrating an indenter at the tip of a needle one could not only quantify between layers of different stiffness, but also navigate to the target location and perform a minimally invasive measurement based on the tissue mechanical properties. An example of an *in situ* AFM indenter for arthroscopic knee cartilage inspection was presented by Imer et al. (2006). The indenter consists of an extensive stabilization stage connected to a piezoelectric scanning module, both of which are inserted into the sample, resulting in a large footprint. Moreover, the lack of calibration of the piezoelectric tube hampered a quantitative analysis.

Here, we demonstrate a ferrule-top indenter on the distal end of a rigid needle and we show its ability to quantify local mechanical properties of tissue *in situ*. Thanks to the remote actuation of the sensor by a piezoelectric translator the size of the needle is limited to the dimensions of the indentation probe at the tip. The performance of our indenter is tested on an engineered layered sample as well as on biological tissue.

## Experimental section

### The ferrule-top force transducer

**Fig. 1:**
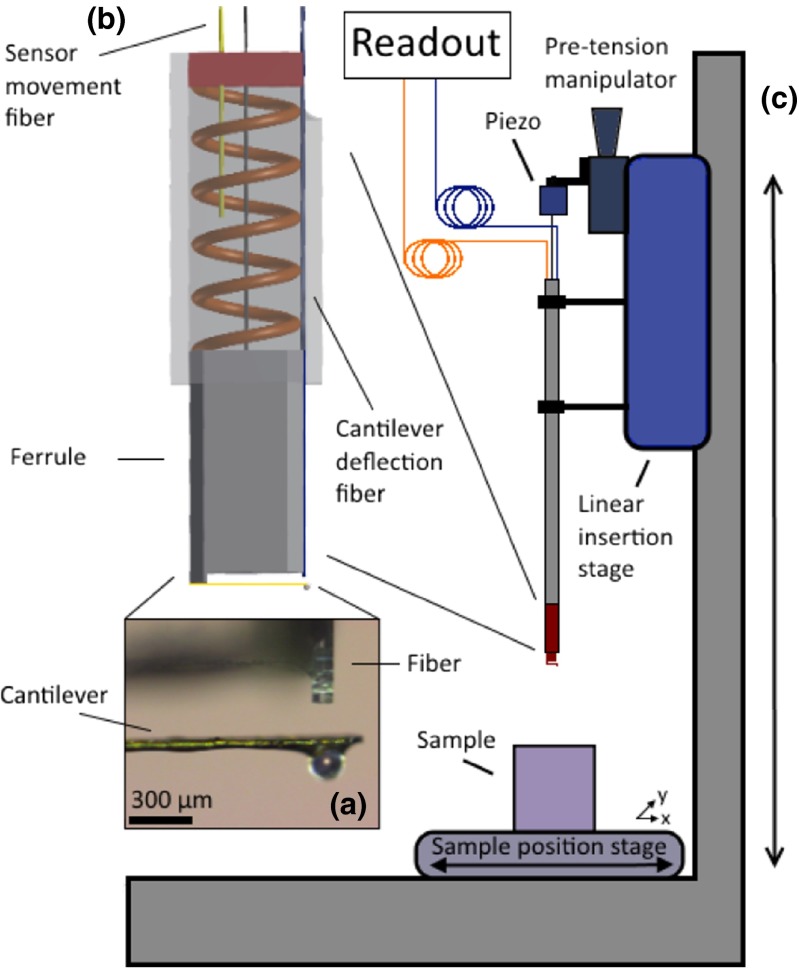
Schematic view of the experimental setup, showing a microscope image of the indenter (**a**), a closeup of the indentation module (**b**), and a sketch of the complete needle insertion setup (**c**) (not to scale)

The indenter relies on the working principle of bulkier ferrule-top instruments, which is here briefly discussed (for more details, see Chavan et al. (2012); Beekmans and Iannuzzi (2015); van Hoorn et al. (2016). A ferrule-top force transducer consists of a borosilicate cantilever, equipped with a small sphere on its free hanging end (see Fig. 1a), and assembled over a 3 mm x 3 mm x 7 mm borosilicate ferrule (see Fig. 1b). A single mode optical fiber is glued at the side of the ferrule and aligned with the free hanging end of the cantilever. The cleaved end of the fiber and the bottom surface of the cantilever form a Fabry-Pérot cavity (more details in Section 2.4), which allows one to detect the bending of the cantilever with nanometer precision. Pushing the sphere of the cantilever against a sample, and measuring the indentation depth as a function of the deflection of the cantilever (and, hence, of the force applied), one can infer the Young Modulus of the sample.

### Indentation module

To reduce the dimensions of the indenter, we have developed an indentation module that enables remote actuation of the force transducer. A schematic view of the indentation module is shown in Fig. 1b. The optical force transducer is housed in a square borosilicate capillary with an inner lumen of 3.05 mm x 3.05 mm, which restricts the movement of the probe to the axial direction. The probe is mechanically connected to a calibrated piezoelectric translator via a steel cable (diameter = 120 *μ*m) similar to those that are commonly used to actuate the tip of surgical steerable needles (Breedveld et al. 2005; van de Berg et al. 2015). A small compression spring is used to load the probe against a backplane in the capillary. If the spring is initially compressed with pretension, in fact, the movement of the piezoelectric translator can be smoothly transferred, from remote position, to the probe. The translator (P-602.5L8, Physike Instrumente GmbH) has a 500 *μ*m stroke and a 325 N blocking force. It is important to note that, due to hysteresis in the steel cable and friction between the capillary and the probe, in our case, it is not possible to assume that the movement of the probe is exactly equal to that indicated by the strain gauge feedback system of the driving piezoelectric device. To solve this issue, the movement of the probe is monitored by a second single mode optical fiber, anchored to the backplane of the square capillary and aligned with the back of the ferrule (see Fig. 1b).

### Experimental setup

The indentation module is housed at the distal end of a 20cm long custom designed needle, that is used to insert the sensor into the specimen. Fig. 1c shows a schematic view of the ferrule-top indenter at the tip of the rigid needle. The needle (diameter = 5mm) is fixed to a motorized linear translation stage (LTS300, Thorlabs GmbH) that is used for insertion in the sample. At the proximal end of the needle the steel cable is fixed to the piezoelectric translator, which in turn is mounted on a coarse position stage, allowing for adjustment of the pretension in the cable-spring system. The two optical fibers (i.e., the cantilever deflection readout and the sensor movement readout), are fed through the lumen of the needle and connected to two interferometric readout systems (OP1550, Optics11)^1^. Both interferometers are equipped with a tunable infrared laser (35 nm tunability), the wavelength of which is internally locked with 10 pm accuracy by a feedback system, and can be swept by driving the injection current sinusoidally.

The sample is placed on an xy-translation stage (MAX312D, Thorlabs GmbH), which is used to select a position for needle insertion. To reduce vibrations, the setup is built on a passive anti-vibration stage.

### Working principle

**Fig. 2:**
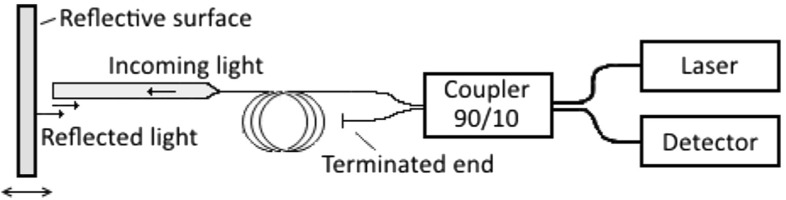
Sketch of the interferometric scheme used to measure the deflection of the cantilever and the movement of the ferrule. The cavity formed by the cleaved end of the fiber and the surface in front is interrogated by a monochromatic tunable laser. The difference in intensity due to a change in interference (caused by movement of the mirror) is detected in the photodiode

As previously stated, both the detection of cantilever bending and the monitoring of the position of the sensor rely on Fabry-Pérot interferometry. Figure 2 illustrates the basic scheme of the detection mechanism that is employed. The laser is coupled into the Fabry-Pérot cavity via the 10 % arm of a 90/10 fiber coupler. Before entering the cavity, a small fraction of the incident light is coupled back into the fiber due to the refractive index mismatch between the fiber core and the medium inside the cavity. The remainder of the light passes through the cleaved fiber end, reflects on the other surface of the cavity (in our case, either the bottom of the cantilever or the bottom of the ferrule) and is collected back into the fiber. The amplitude of the interference pattern created by the two backpropagating signals is measured by a photodiode aligned with the exit of the coupler and can be described by^2^:

1 $$ W(d)=W_{0} \left[1+V \cos\left( \frac{4 \pi d}{\lambda}+ \varphi_{0} \right)\right], $$

where *d* is the length of the cavity, *λ* is the central wavelength of the laser, *φ*_0_ is a constant phase shift that only depends on the geometry of the probe, and *W*_0_ and *V* are the midpoint interference signal ((*W*_*max*_ + *W*_*min*_)/2) and the fringe visibility ((*W*_*max*_−*W*_*min*_)/(*W*_*max*_ + *W*_*min*_)), respectively. One can recognize that a displacement of the mirror can be immediately identified from the output signal of the photodiode. In our setup, this principle is employed to assess the position of both the cantilever and the sensor (see Fig. 1). One single mode fiber is positioned perpendicular to the gold coated bottom facet of the cantilever and a second fiber is anchored at the backplane of the square capillary, continuously monitoring the displacement of the sensor. Multiple reflections in the cavity can be prevented by a slight rounding of the cleaved facet of the fiber, obtained by exposing the tip to a brief plasma arc before mounting it in the setup.

The detection method described is highly sensitive for relative mirror movements and is straightforward to implement for small displacements. One can simply tune the wavelength to quadrature, where the sensitivity to mirror movement is maximum and the output of the readout is linear. Although effective for small displacements around quadrature, the non-linearity of the output signal renders the method not ideal for the larger displacements required for indentation measurements, where a linear readout is required over a large displacement (*d*≫*λ*). In order to linearize the amplitude response over the complete deflection range we modulated the wavelength of the laser around the central wavelength (*λ*_*c*_) according to van Hoorn et al. (2016):

2 $$ \lambda (t) = \lambda_{c} + \delta\lambda \cos(\omega t), $$

where *δλ* and *ω* represent the amplitude and frequency of the modulation, respectively. Assuming, for the sake of simplicity and without loss of generality, *φ*_0_ = 0, the expected time dependent amplitude of the photodiode during wavelength modulation is given by:

3 $$ W(t) = W_{0} \left( 1+V\cos\left[\frac{4 \pi d}{\lambda_{c} +\delta\lambda \cos\left( \omega t\right)}\right]\right). $$

This time dependent response contains a DC component, encoding for the movement of the reflective surface, and a component that oscillates at frequency *ω*, originating from the modulation of the wavelength. The contribution of each component can be assessed in detail by making a first order Taylor expansion of *W*(*t*) around $\frac {\delta \lambda \cos ?\left (\omega t\right )}{\lambda _{c}}=0$:

4 $$ W(t) \approx \cos\left( \frac{4 \pi d}{\lambda_{c}}\right) + \left( \frac{4 \pi d \delta\lambda \cos\left( \omega t\right)}{{\lambda_{c}^{2}}}\right) \sin\left( \frac{4 \pi d}{\lambda_{c}}\right). $$

The low frequency component (which we will denote with *W*_*dc*_) and the high frequency component (which we will denote with *W*_*ω*_) are now described by the first and second term of the Taylor expansion, respectively. It can be readily observed from Eq. 4 that *W*_*dc*_ and *W*_*ω*_ are separated by a 90 deg phase shift. This particular relation allows one to linearize the output signal and apply phase unwrapping to obtain a continuous linear response for the displacement of the mirror (*D*_*m*_):

5 $$ D_{m} = \frac{\lambda_{c}}{4 \pi} \arctan(W_{dc}/W_{\omega}). $$

*W*_*dc*_ can then be recorded by means of a low-pass filter with a cut-off frequency below the modulation frequency. To record *W*_*ω*_, the unfiltered amplitude response of the photodiode is sent to a lock-in amplifier, which is locked at frequency *ω* via a square wave reference signal. We note that, thanks to the high bandwidth of our measurement, acquisition of *W*_*dc*_ and *W*_*ω*_ and the following linearization of the signal is performed in real time.

### Specimen preparation

To demonstrate the working principle of our device, we performed a series of insertions consisting of multiple indentation measurements at varying depth on two gelatin-based phantoms (Gelatin from bovine skin, Sigma-Aldrich). In the first specimen a stiffness gradient was created by compiling layers (10 mm in height) with decreasing mass gelatin to water ratio, hence creating a sample with layers of decreasing stiffness from bottom to top. Starting from a layer of 15 % mass gelatin at the bottom, each following layer contained 2.5 % less mass gelatin, ultimately creating a specimen with 6 layers of decreasing stiffness: 15 %, 12.5 %, 10 %, 7.5 %, 5 % and 2.5 % mass gelatin. For each layer, the gelatin was dissolved in demineralised water at 60 ° C, poured in the container and kept at 4 ° C for 30 minutes to allow the layer to stiffen. After the final layer was poured, the sample was stored 4 ° C overnight and measured the next day.

The second specimen consisted of a square piece of bovine liver (6x6x3 mm ^3^) fixated in a gelatin solution. To create a base layer (20 mm in height), gelatin was dissolved at a 12 % mass to water ratio at 60 °C, poured in a container and stored overnight at 4 °C. Subsequently, the liver sample was placed on top of the first gelatin layer and a second solution of gelatin (12%) was poured over the sample until it was fully submerged. The gelatin solution was cooled down to 40 °C prior to pouring to prevent thermal damage of the tissue. After the container was filled with gelatin (± 20 mm above the sample), it was again stored overnight at 4 °C to ensure proper stiffening.

### Experimental details and indentation protocol

For this experiment, the cantilever was equipped with a spherical borosilicate bead of radius equal to 85 *μ*m. For optimal sensitivity, the cleaved end of the single mode fiber was positioned directly underneath the center of the microbead, positioning the Fabry-Pérot cavity directly underneath the point of contact (Fig. 1a). A central wavelength of 1551 nm was used in both interferometers in combination with a 90 kHz sinusoidal wavelength modulation of approximately 50-200 pm modulation depth. The spring constant of the cantilever was measured to be 12.97 ± 0.06 N/m via the method reported in Beekmans and Iannuzzi (2015).

Four needle insertions were placed in the layered specimen and six in the animal liver specimen. Stiffness data were recorded at seven depth: one at the surface and six at increasing depth, each position separated by 10 mm. At each point in depth 5 indentation curves were recorded at 3 different locations, for a total of 15 indentations per depth position. All the measurements were performed in air in order to best preserve the gelatin structure. For each depth level, the probe at the end of the needle was carefully lowered with the motorized linear stage until contact was found between the indenting tip of the ferrule-top probe and the sample, identified by backwards bending of the cantilever of a few tens of nanometers. Starting from this position, the indentation movement was generated by applying a ramp-shaped voltage profile to the piezoelectric translator. This movement was in turn translated through the needle shaft and resulted in an indentation stroke of the sensor. Prior to each measurement, the system was adjusted such that the stroke of the sensor (*d*_*f*_) was maximum 30 *μ*m. The indentation depth (*d*_*i*_) inside the sample, however, depends not solely on *d*_*f*_ but also on the displacement cantilever (*d*_*c*_). The indentation depth was continuously recorded during the measurement and is given by:

6 $$ d_{i}=d_{f}-d_{c}. $$

If the spring constant of the cantilever (*k*) is known, the load (*P*) applied during the indentation is:

7 $$ P= k \cdot d_{c}. $$

To validate our measurements, before the first insertion of the needle, we cut a cross-sectional slice of the specimen, in which the different layers were clearly identifiable. The slice was then indented on the surface at locations corresponding to the different layers. Each reference measurement was obtained as an average of 15 indentations spread over 3 locations.

### Analysis

**Fig. 3:**
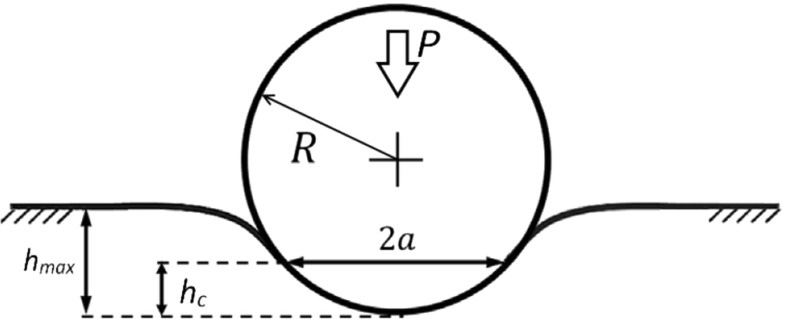
Schematic view of the elastic indentation of a flat plane surface with a small spherical indenter of radius *R*. Starting from zero load (*P* = 0), *P* is increased until the maximum indentation depth (*h*_*max*_) in reached. The contact radius (*a*) and the corresponding contact depth (*h*_*c*_) depend on the depth of indentation

In order to derive the elastic modulus from the raw indentation data, we used the method for spherical indentation described by Oliver and Pharr (1992); Oliver and Pharr (2004). It is important to recall that, to describe the indentation of an elastic material with a spherical indenter the contact area *A* between the tip and the sample must be correctly modeled. The contact depth (*h*_*c*_) of the sphere can be determined from the final and maximum indentation depths (*h*_*f*_ and *h*_*max*_, respectively) (Field and Swain 1993) (Fig. 3):

8 $$ h_{c}= \frac{h_{max}+h_{f}}{2}. $$

Now, from a geometrical point of view, the radius of the circle of contact can be calculated from:

9 $$ a = \sqrt{(2Rh_{c} - {h_{c}^{2}})}, $$

where *R* is the radius of the spherical tip. For a contact depth significantly smaller than the radius of the indenter the quadratic term in the square root can be neglected. Assuming initial elastic unloading, the Young Modulus of the indented material can be estimated from the experimental data by Hertzian mechanics:

10 $$ E=\frac{S\sqrt{\pi}}{2\sqrt{A}}(1-\nu^{2}). $$

Here, *S* = *dP*/*dh* is the slope of the initial unloading curve (i.e., 85 % and 65 % of the load at maximum indentation), *A* = *πa*^2^ is the area of the contact circle and *ν* is the Poisson ratio of the indented material. In our case *ν* = 0.5, as both gelatin and liver tissue were assumed to be incompressible (Humphrey 2003; Fung 1993).

## Results and discussions

**Fig. 4:**
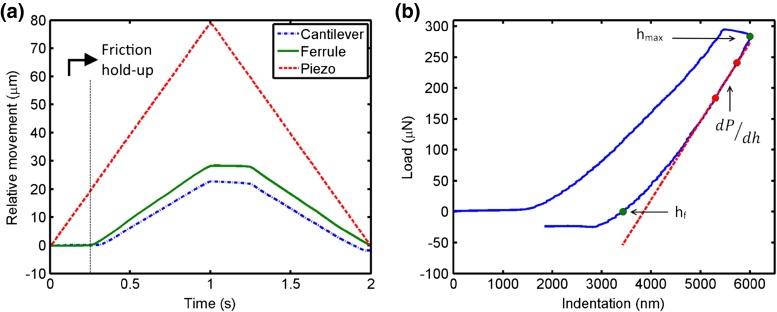
**a**) Relative movement of the piezoelectric translator (*red*), of the ferrule, and of the cantilever as a function of time in one of the indentation stroke. The micro-movement of the sensor (*green*) and the cantilever (*blue*) at the tip of the needle is compared with the movement of the piezoelectric transducer at the proximal end. The remote actuation of the ferrule results in a smooth, reliable indentation movement. The pretension of the spring is adjusted such that the hold time at maximum load is 250 ms. **b**) Load-indentation curve obtained from the data reported in A, along with the definition of the parameters used for the analysis of the data

The response of the ferrule-top probe to a ramp-like movement of the piezoelectric transducer as observed in one of our indentation stroke is reported in Fig. 4a. One can note that, at the start of the ramp, the friction of the probe inside the indentation module prevents the probe from moving. However, as the force builds up the friction is overcome and a smooth, reliable indentation movement is initiated. We observed that the amount of friction (and the hereby related hold time) is adjustable by varying the initial loading of the compression spring. Using our method, we were able to remotely actuate the indenter with the precision required for our indentation measurement. Fig. 4b reports a typical load versus indentation depth curve obtained with our system. Note that indentation in our case describes the deformation of the measured sample, not the deflection of the cantilever.

**Fig. 5:**
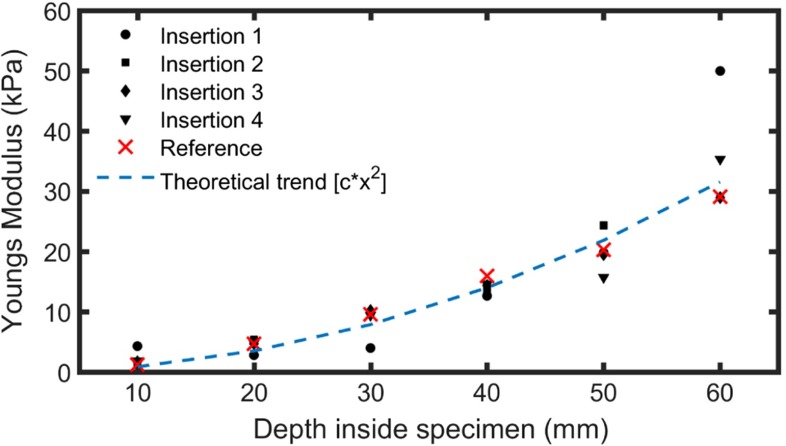
Young Modulus versus depth in the gelatin gradient specimen for 4 insertions (*closed circles, squares, diamonds, triangles*) compared to surface measurements on a cross-sectional reference slice (*crosses*). The *dashed line* was added as a guide for the eye. Still, it is to note that that line scales like the square power of the depth, which corresponds to the trend expected from theory (Hall et al. 1997)

In Fig. 5, we compare the Young Moduli measured during four separate needle insertions in the gelatin gradient specimen with the results obtained from the reference slice. One can conclude that there is a good agreement between the two measurements, although some outliers are, at times, observed. Some of those outlying data may be due to the presence of contaminations on the measured surface. The results obtained at 60 mm depth, on the contrary, can be probably ascribed to the fact that the bottom layer was too close to the bottom of the glass container.

**Fig. 6:**
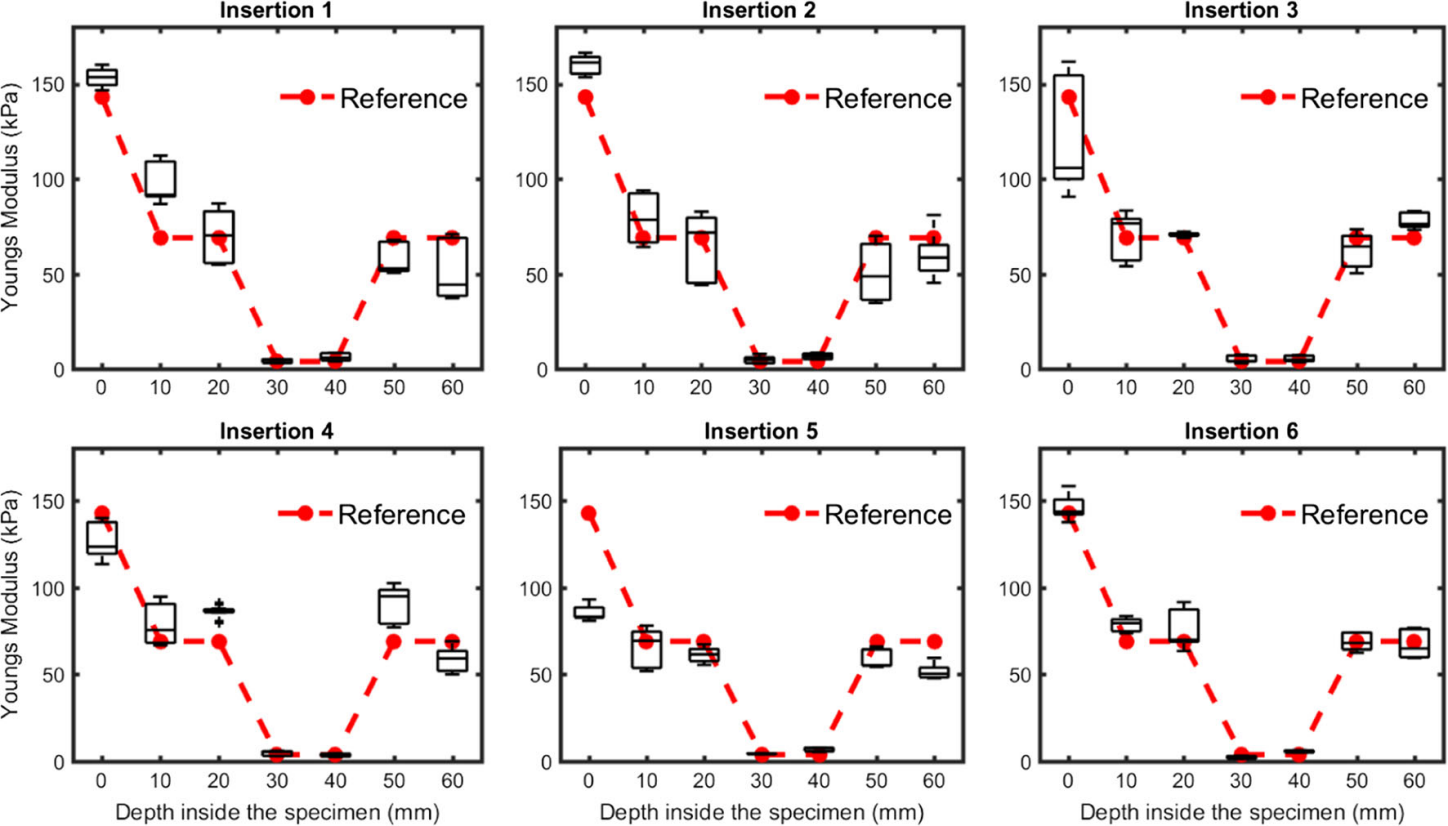
Boxplot of Young Modulus versus depth in the fixed liver specimen for six needle insertions, compared with a cross-sectional reference (*dashed line*). The lever slide was placed 25 mm underneath the surface

**Fig. 7:**
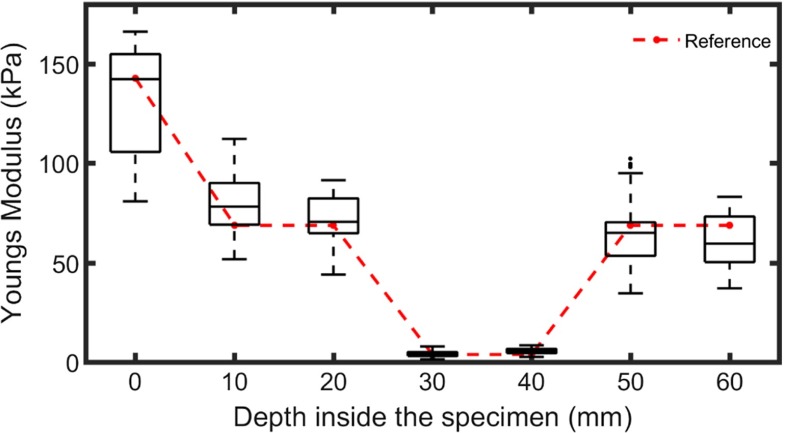
Boxplot of the grouped Young Moduli versus depth in the liver specimen for all six indentations, compared with the cross-sectional reference (*dashed line*)

Figure 6 shows the Young Modulus at increasing depth for six insertions in the fixed liver specimen and the corresponding cross-sectional reference. As one can see from the reference, the specimen consisted of thee different stiffness levels; 1) the surface of the specimen, 2) the encapsulating gelatin and 3) the liver. The surface of the specimen was found to be noticeably stiffer than the gelatin underneath, most likely due to the evaporation of water that occurs when the specimen is in contact with air. The liver, one order of magnitude softer than the surrounding gelatin, was positioned between 25 and 45 mm from the surface. The difference in stiffness between gelatin and the liver tissue is clearly visible in the reference and for each insertion. The homogeneous stiffness distribution of the gelatin over the entire specimen (with exception of the surface), as demonstrated by the reference measurement, is less predominant when each insertion is inspected individually. This variability can be attributed to the inhomogeneity of the measured surface during the insertion; surface roughness can cause the contact to be ill-defined and can lead to an under- or overestimation of the sample stiffness. However, when all the insertions are grouped together (Fig. 7), a good agreement is observed between the gelatin measurements at different depths, as well as between the liver indentations at 30 and 40 mm.

## Limitations

In the following section we discuss some of the limitations of our indenter in its current form.

The design of the cantilever for this experiment was optimized to measure the stiffness of materials with Young Modulus between 1 kPa and 100 kPa. To cover the wide range of biological tissue stiffness, which varies over at least three orders of magnitude (McKee et al. 2011), one may need to use cantilevers with different spring constants – a major complication for future applications. Furthermore, throughout the entire analysis, we have implicitly neglected the viscous and plastic components of the sample mechanics. Both these issues have been recently addressed by another paper of our group (van Hoorn et al. 2016), where we showed that, applying a dynamic modulation analysis, it is possible to measure both the loss and storage modulus of a largely heterogenous material. Still, the method adds some limitations, as it is not as fast and as straightforward as the one presented here.

A further obvious limitation of our needle indenter here is its large diameter. Work is under way to reduce the dimensions of all its components to embed the device in a 3 mm diameter needle.

Another possible drawback is the risk that, during perforation, some debris of the sample enters the Fabry-Pérot cavity between the fiber and the cantilever. Although we have not observed any nuisance when indenting inside the liver specimen, one could circumvent this problem altogether by designing a membrane based sensor.

Finally, for further research on biological samples, sterilization of the device may become necessary. We designed the device such that the sensitive part, which will not survive a repetitive sterilization procedure, can be disposed without discarding the main working elements of the indenter.

## Conclusions

We have successfully developed a cantilever based, all-optical indenter at the tip of a rigid needle. The indentation measurement is enabled by a sensor that probes the mechanical properties of the underlying specimen by indentation using a microsphere. The sensor is remotely actuated by a strain gauge controlled piezoelectric translator driving a microscopic cable and spring system. The movement of the sensor as well as the movement of the cantilever is interrogated by Fabry-Pérot interferometry. We have performed stiffness measurements at fixed depth positions during needle insertion in gelatin phantoms and animal liver specimens. The measurements showed that we are able to quantify a stiffness gradient in depth and that we can successfully identify stiffer layers in a uniform sample. Measurements in a gelatin embedded animal liver confirmed that we can localize the liver based on mechanical contrast. Moreover, as the needle protrudes further inside the liver tissue, a quantitative analysis of the liver can be made based on the mechanical properties alone. Despite some limitations, our needle may, on the long term, ultimately evolve into a minimally invasive tool for the analysis of the mechanical properties of tissues, with potential applications in needle navigation or tissue diagnostics.

## Footnotes

1 Declaration of interest: D. Iannuzzi is shareholder of Optics11.

2 Mode hopping in the laser is minimized by introducing an isolator in the optical path.

## References

[CR1] Beekmans SV, Iannuzzi D (2015). Surf. Topogr.: Metrol. Prop..

[CR2] Breedveld P, Sheltes J, Blom E, Verheij J (2005). IEEE Eng. Med. Biol. Mag..

[CR3] Butler DL, Goldstein SA, Guilak F (2000). J. Biomech. Eng..

[CR4] Chavan D, Watering TCVD, Gruca G, Rector JH, Heeck K, Slaman M, Iannuzzi D (2012). Rev. Sci. Instrum..

[CR5] S.C. Cowin, S.B. Doty, Tissue Mechanics (Springer Science & Business Media (2007)

[CR6] S.C. Cowin, J.D. Humphrey, Cardiovascular Soft Tissue Mechanics (Springer Science & Business Media (2007)

[CR7] Desrochers J, Amrein MA, Matyas JR (2010). Journal of Biomechanics.

[CR8] Draaijers LJ, Botman YAM, Tempelman FRH, Kreis RW, Middelkoop E, van Zuijlen PPM (2004). Burns.

[CR9] Durani P, McGrouther DA, Ferguson MWJ (2009). J. Plast. Reconstr. Aesthet. Surg..

[CR10] Field J, Swain M (1993). J. Mater. Res..

[CR11] Franze K (2011). Curr. Opin. Genet. Dev..

[CR12] Friedl P, Wolf K. (2003). Nat. Rev. Cancer..

[CR13] Fu J, Wang YK, Yang MT, Desai RA, Yu X, Liu Z, Chen CS (2010). Nat. Meth..

[CR14] Fung YC (1993). Biomechanics.

[CR15] H.O.B. Gautier, A.J. Thompson, S. Achouri, D.E. Koser, K. Holtzmann, E. Moeendarbary, K. Franze, Vol. 125, ed. by E.K. Paluch. *In: Methods in Cell Biology Biophysical Methods in Cell Biology* (Academic Press, 2015), pp. 211–235. http://www.sciencedirect.com/science/article/pii/S0091679X14000065 10.1016/bs.mcb.2014.10.005 25640431

[CR16] Gurtner GC, Werner S, Barrandon Y, Longaker MT (2008). Nature.

[CR17] Hall T, Bilgen M, Insana M, Krouskop T (1997). IEEE Trans. Ultrason. Ferroelectr. Freq. Control.

[CR18] Hengsberger S, Kulik A, Zysset P (2001). Eur. Cell Mater,.

[CR19] Huang H, Kamm RD, Lee RT (2004). Am. J. Physiol. Cell Physiol..

[CR20] Humphrey JD (2003). Proceedings of the royal society of london A: mathematical. Phys. Eng. Sci..

[CR21] Imer R, Akiyama T, de Rooij NF, Stolz M, Aebi U, Friederich NF, Koenig U, Wirz D, Daniels AU, Staufer U (2006). Jpn. J. Appl. Phys..

[CR22] Kahn JS, Trifonov A, Cecconello A, Guo W, Fan C, Willner I (2015). Nano. Lett..

[CR23] Li QS, Lee GYH, Ong CN, Lim CT (2008). Biochem. Biophys. Res. Commun..

[CR24] Li M, Liu LQ, Xi N, Wang YC (2015). Acta Pharmacol. Sin..

[CR25] Mathur AB, Collinsworth AM, Reichert WM, Kraus WE, Truskey GA (2001). J. Biomech..

[CR26] McKee CT, Last JA, Russell P, Murphy CJ (2011). Tissue Eng. Part B Rev..

[CR27] Murphy MC, Huston J, Jack CR, Glaser KJ, Manduca A, Felmlee JP, Ehman RL (2011). J. Magn. Reson. Imaging.

[CR28] Neufurth M, Wang X, Tolba E, Dorweiler B, Schröder HC, Link T, Diehl-Seifert B, Müller WEG (2015). PLoS ONE.

[CR29] Oliver W, Pharr G (1992). J. Mater. Res..

[CR30] Oliver W, Pharr G (2004). J. Mater. Res..

[CR31] Plodinec M, Loparic M, Monnier CA, Obermann EC, Zanetti-Dallenbach R, Oertle P, Hyotyla JT, Aebi U, Bentires-Alj M, Lim RYH, Schoenenberger CA (2012). Nat. Nano..

[CR32] Stolz M, Gottardi R, Raiteri R, Miot S, Martin I, Imer R, Staufer U, Raducanu A, Düggelin M, Baschong W, Daniels AU, Friederich NF, Aszodi A, Aebi U (2009). Nat. Nano..

[CR33] Streitberger KJ, Sack I, Krefting D, Pfüller C, Braun J, Paul F, Wuerfel J (2012). PLoS ONE.

[CR34] Swift J, Ivanovska IL, Buxboim A, Harada T, Dingal PCDP, Pinter J, Pajerowski JD, Spinler KR, Shin JW, Tewari M, Rehfeldt F, Speicher DW, Discher DE (2013). Science.

[CR35] van de Berg N, van Gerwen D, Dankelman J, van den Dobbelsteen J (2015). IEEE/ASME Trans. Mechatron..

[CR36] H. van Hoorn, N. Kurniawan, G. Koenderink, D. Iannuzzi, Submitted for publication (2016) 10.1039/c6sm00300a PMC4819682 26908197

[CR37] Wuerfel J, Paul F, Beierbach B, Hamhaber U, Klatt D, Papazoglou S, Zipp F, Martus P, Braun J, Sack I (2010). NeuroImage.

[CR38] Zhu Y, Dong Z, Wejinya UC, Jin S, Ye K (2011). J. Biomech..

